# Buruli ulcer treatment: Rate of surgical intervention differs highly between treatment centers in West Africa

**DOI:** 10.1371/journal.pntd.0007866

**Published:** 2019-10-28

**Authors:** Anita C. Wadagni, Jonathan Steinhorst, Yves T. Barogui, P. M. Catraye, Ronald Gnimavo, Kabiru M. Abass, George Amofa, Michael Frimpong, Francisca N. Sarpong, Tjip S. van der Werf, Richard Phillips, Ghislain E. Sopoh, Christian R. Johnson, Ymkje Stienstra

**Affiliations:** 1 University of Groningen, University Medical Center Groningen, Department of Internal Medicine/Infectious Diseases, Groningen, The Netherlands; 2 Programme National de Lutte contre la Lèpre et L'Ulcère de Buruli, Ministère de la Santé, Cotonou, République du Bénin; 3 Agogo Presbyterian Hospital, Agogo, Ghana; 4 Dunkwa Government Hospital, Dunkwa, Ghana; 5 Kwame Nkrumah University of Science and Technology (KNUST), School of Medical Sciences and Kumasi Centre for Collaborative Research in Tropical Medicine (KCCR), Kumasi, Ghana; Swiss Tropical and Public Health Institute, SWITZERLAND

## Abstract

**Background:**

Antibiotic treatment proved itself as the mainstay of treatment for Buruli ulcer disease. This neglected tropical disease is caused by *Mycobacterium ulcerans*. Surgery persists as an adjunct therapy intended to reduce the mycobacterial load. In an earlier clinical trial, patients benefited from delaying the decision to operate. Nevertheless, the rate of surgical interventions differs highly per clinic.

**Methods:**

A retrospective study was conducted in six different Buruli ulcer (BU) treatment centers in Benin and Ghana. BU patients clinically diagnosed between January 2012 and December 2016 were included and surgical interventions during the follow-up period, at least one year after diagnosis, were recorded. Logistic regression analysis was carried out to estimate the effect of the treatment center on the decision to perform surgery, while controlling for interaction and confounders.

**Results:**

A total of 1193 patients, 612 from Benin and 581 from Ghana, were included. In Benin, lesions were most frequently (42%) categorized as the most severe lesions (WHO criteria, category III), whereas in Ghana lesions were most frequently (44%) categorized as small lesions (WHO criteria, category I). In total 344 (29%) patients received surgical intervention. The percentage of patients receiving surgical intervention varied between hospitals from 1.5% to 72%. Patients treated in one of the centers in Benin were much more likely to have surgery compared to the clinic in Ghana with the lowest rate of surgical intervention (RR = 46.7 CI 95% [17.5–124.8]). Even after adjusting for confounders (severity of disease, age, sex, limitation of movement at joint at time of diagnosis, ulcer and critical sites), rates of surgical interventions varied highly.

**Conclusion:**

The decision to perform surgery to reduce the mycobacterial load in BU varies highly per clinic. Evidence based guidelines are needed to guide the role of surgery in the treatment of BU

## Author summary

Buruli ulcer is a necrotizing and disabling skin infection, caused by *Mycobacterium ulcerans*. The infection, a skin-related Neglected Tropical Diseases, affects mostly people living in limited resources settings. Since the introduction of rifampicin based combination antibiotic therapy as standard care, the role of surgery as adjunct therapy *to kill M*. *ulcerans* is less defined and understood. A randomized controlled trial showed benefit from delaying the decision to operate. Nevertheless, the rate of surgical interventions differs highly per clinic. We present the differences in rate of surgical interventions in six different Buruli ulcer treatment centers in Ghana and Benin. We demonstrate that these differences mainly depend on the opinion of the health care workers working in the treatment centers even after adjusting for disease severity.

## Introduction

Buruli ulcer (BU), is a neglected tropical disease caused by *Mycobacterium ulcerans*. The disease mostly affects poor people in rural areas with limited access to health care. It can manifest as a nodule, a plaque or an oedematous lesion that can progress, in the absence of treatment, to a large ulcer and/or long-term functional disability [1]. Before 2004, the most effective treatment for BU was by extensive surgery. Then, antibiotic treatment was added gradually [2].

The current WHO guidelines for BU treatment consist of a combination of antibiotics (rifampicin /clarithromycin) and, if necessary, surgical intervention. Surgical treatment consists of excision or debridement followed by skin grafting [3,4]. Buruli ulcer is frequently characterized by a paradoxical response after start of antimicrobial treatment. These paradoxical reactions manifest as a deterioration in the clinical appearance of the lesion and they can be misinterpreted as failure to respond to treatment, leading to unnecessary surgical intervention [5]. The current WHO protocol for BU treatment advises to decide whether surgery is needed four weeks after the start of antimicrobial treatment but guidelines for the type and timing of surgical intervention in an African setting are not completely defined [2,3]. Some consider early surgical intervention necessary to prevent disabilities and contracture deformities [2,6]. However, different clinical trials have proven that antimicrobial treatment can effectively cure both early lesions and severe lesions and postponing the decision for surgical intervention leads to less surgical interventions needed without a difference in treatment outcomes including long-term disabilities [7,8]. In Australia, the decision on the combination of oral antibiotics and surgery is different, influenced by the widely available surgical resources and the earlier stage of disease of patients presenting with [6,9,10]. But in less resourced settings, surgical intervention represents a burden to the health system due to its costs and fear of surgery was earlier reported by patients to be one of the reasons for patients’ delay [11].

We studied the differences in rate of surgical interventions per treatment center in addition to the antimicrobial therapy to explore whether these differences are mainly caused by different patient populations or mainly by expert opinion of the health care workers working in the treatment centers.

## Method

### Study design and population

This was a retrospective study conducted in six different Buruli ulcer treatment centers. Former clinically diagnosed BU patients from Benin and Ghana were identified using the Benin National Buruli Ulcer Control Program database and medical records kept by the clinics in Ghana. The study was conducted in three hospitals in Benin (Centre de Dépistage et de Traitement de l'Ulcère de Buruli (CDTUB) in Lalo, Centre de Dépistage et de Traitement de l'Ulcère de Buruli CDTUB in Allada, Centre de Diagnostic et de Traitement de l'Ulcère de Buruli CDTUB de Pobè) and three clinics in Ghana (Agogo Presbyterian Hospital, Tepa Government Hospital and Dunkwa Hospital). Patients were eligible for analysis if they were clinically diagnosed as BU patients in accordance with the WHO case definition [1] and/or, if diagnostic tests were not performed, successfully received full antimicrobial treatment for BU, suggesting *M*. *ulcerans* as the etiological factor of their illness. Patients were included if they received the diagnosis between 1 January 2012 and 31 December 2016. Patients reported at a later date were excluded due to the concern that treatment for BU may require surgical intervention up to one year after start of antimicrobial treatment. Patients presenting with a recurrence, or who were unresponsive to treatment, or found to have an alternative diagnosis later during BU treatment were excluded from the analysis. Patients recruited in CDTUB of Allada before September 2014, and those recruited in CDTUB of Lalo before December 2015 were excluded due to the randomized clinical trial on surgical intervention conducted between July 2011 to December 2015 in Lalo and July 2011 to September 2014 in Allada [8]. Data was collected from February to March 2018 in Ghana and between July and October 2018 in Benin. Data was collected from WHO BU01 and BU02 forms, patient medical records, lab confirmation slips and operating theatre records at the respective treatment centers.

The hospitals in Lalo, Allada, Pobè and the clinics in Agogo and Dunkwa had the capacity to perform surgery at their own facility. The hospital in Tepa referred patients for surgical intervention to Agogo Presbyterian Hospital.

### Study parameters

To describe socio-demographic and clinical characteristics of the study population, the following variables were sampled: age, gender, type of lesion (nodule, plaque, oedema or ulcer), WHO category (category I, II or III), location of lesion (upper limb, lower limb, others), lesions at critical sites (eyes, breast, genital parts), functional limitations at diagnosis, and surgery performed during treatment.

In terms of severity, WHO has classified BU into three categories. Category I lesions are single small lesions e.g. nodules, papules, plaques, and ulcers less than 5 cm in diameter, Category II lesions consist of non-ulcerative or ulcerative plaques, edematous forms, single large ulcerative lesion of 5–15 cm in cross-sectional diameter. Category III lesions are either at critical sites—notably, the face, breast and genitals; or disseminated and mixed forms including osteomyelitis, and extensive lesions of more than 15 cm [3].

Surgical intervention included procedures such as excision and debridement (if performed in theatre) that were intended to reduce the mycobacterial load and therefore contribute to the sterilization of the lesion. Bedside removal of necrotic tissue and skin grafting were not considered as surgery intended to affect *M*. *ulcerans* itself. The need for skin grafting was recorded but not included in the analysis answering the research question on the need of surgical intervention, as skin grafting is typically not intended to reduce the mycobacterial load but only to prevent delayed healing with complicating scar formation, contractures and movement restrictions.

### Data analysis

Data was analyzed using SPSS version 24 (IBM). General descriptives were reported as median (IQR 25–75) and frequency (%) per country. The differences in the rate of surgery were compared across clinics, and between patients according to whether or not they presented with functional limitations, or severe lesions (ulcer, critical site) using Pearson chi-square, or Fischer exact test as appropriate. The center with the lowest rate of surgical interventions was used as reference to compare surgical practices with the other BU treatment centers. Binary logistic regression analysis, using a manual backward elimination procedure, was carried out to estimate the effect of the treatment center on the outcome measure surgical intervention, while checking for interaction (severity of disease) and confounders (severity of disease, age, gender, limitation of movement at joint at time of diagnosis, ulcer and critical sites). The rate of surgical interventions was compared over the years using Linear by linear Association.

### Ethics statement

The subjects of the study are made anonymous by the assignment of identification numbers to protect the privacy of patients and the confidentiality of personal information.

Ethical clearance for the study was obtained from the medical ethical committee (METc) of the University Medical Center Groningen and the Committee on Human Research, Publication and Ethics of the Kwame Nkrumah University of Science and Technology in Kumasi, Ghana (reference number: CHRPE/AP/468/17) and from the Comité d’Éthique de la Faculté des Sciences de la Santé, Cotonou, Benin (reference number:005-19/UAC/FSS/CER-SS)

## Results

### Study participants

Between 1 January 2012 and 31 December 2016, 1464 medical records of patients with Buruli ulcer were registered at the facilities in Benin (Allada, Lalo, Pobè) and Ghana (Agogo, Dunkwa, Tepa). Of these, 30 patients were excluded because of a recurrence of BU; 10 were identified as not having BU; 4 had surgeries with an unknown type of procedure (skin graft, debridement, or any other surgical intervention); and 227 were former participants of the randomized clinical trial on surgical intervention conducted in Lalo and Allada. A total of 1193 patients, 612 from Benin and 581 from Ghana, were included for the analysis (supporting information 2). PCR was performed in 92% of the patients and was positive for *M*. *ulcerans* in 82% of the patients.

560 patients of the total study population were male (46.9%) and the median age was 14 years (IQR 8–31). All patients received antimicrobial treatment. In Benin, most lesions were category III lesions (42.0%) while in Ghana most lesions were category I lesions (43.7%). There was almost no difference between Benin and Ghana regarding the location of the lesion. In both countries, lesions were mainly located at the lower limb (56.5% in Benin and 57.5% in Ghana). Thirty-seven patients (3.1%) had lesions at critical sites and 282 (23.6%) patients had a functional limitation at time of diagnosis. 161 patients (13.5%) received skin grafts as part of the treatment of the wound. Surgical intervention to remove affected tissue and therefore to treat *M*. *ulcerans* was performed in 344 (28.8%) patients (Table 1).

**Table 1:** Characteristics of patients included in the study.

		Benin (N = 612)	Ghana (N = 581)	Total (N = 1193)
Age in years, Median (IQR)		12 (7–26)	16 (9–36)	14 (8–31)
Sex				
	(Male %)	283 (46.2)	277 (47.7)	560 (46.9)
	Missing	2 (0.3)	2 (0.3)	4 (0.3)
Type of lesions- number of lesions (%)				
	Ulcer	129 (21.1)	297 (51.1)	426 (35.7)
	Nodule	11 (1.8)	137 (23.6)	148 (12.4)
	Plaque	35 (5.7)	80 (13.8)	115 (9.6)
	Oedema	13 (2.1)	23 (4.0)	36 (3.0)
	Osteomyelitis	12 (2.0)	1 (0.2)	13 (1.1)
	Combined pre-ulcerative +ulcerative lesion	410 (67.0)	23 (4.0)	433 (36.3)
	Missing	2 (0.3)	20 (3.4)	22 (1.8)
Category of lesions- number of lesions (%)				
	I	90 (14.7)	254 (43.7)	344 (28.8)
	II	265 (43.3)	171 (29.4)	436 (36.5)
	III	257 (42.0)	118 (20.3)	375 (31.4)
	Missing	0	38 (6.5)	38 (3.2)
Site of lesions- number of lesions (%)				
	Upper Limb	216 (35.3)	191 (32.9)	407 (34.1)
	Lower Limb	346 (56.5)	334 (57.5)	680 (57.0)
	Others	31 (5.1)	27 (4.6)	58 (4.9)
	Multiple	16 (2.6)	26 (4.5)	42 (3.5)
	Missing	3 (0.5)	3 (0.5)	6 (0.5)
Functional limitation (%)				
	Yes	181 (29.6)	101 (17.4)	282 (23.6)
	Missing	33 (5.4)	34 (5.9)	67 (5.6)
Critical sites (%)				
	Yes	11 (1.8)	26 (4.5)	37 (3.2)
	Missing	13 (2.1)	11 (1.9)	24 (2.0)
Surgery (%)				
	Yes	315 (51.5)	29 (5.0)	344 (28.8)

### Surgery

Surgical interventions were performed for patients from all clinics (patients from Tepa had surgery in Agogo). Agogo had the lowest rate of surgical interventions at 1.5% and therefore served as a reference. In our analysis we therefore compared the frequency of operations in other hospitals with this reference. The rate of surgery was 13.2% in Dunkwa (RR = 8.6 CI 95% [3.0–24.4]), 1.9% in Tepa (RR = 1.3 CI 95% [0.3–5.6]), 71.8% in Allada (RR = 46.7 CI 95% [17.5–124.8]), 30.8% in Lalo (RR = 20.0 CI 95% [5.6–71.2]) and 49.2% in Pobè (RR = 32.0 CI 95% [12.1–85.0]). Among patients who presented with a functional limitation at admission, 134 had surgery (47.5%) (RR = 2.0 CI 95% [1.7–2.4] compared to those without functional limitation at admission). Surgery was rare (2.7%) in patients presenting with lesions at a critical site (RR = 0.1 CI 95% [0.01–0.6] compared to those without lesions at a critical site (Table 2).

**Table 2:** Differences in surgical practice between clinics and patient characteristics.

		Surgery		Total	p-value *	RR CI 95%
		Yes	No			
Clinics (%)						
	Agogo	4 (1.5)	256 (98.5)	260		
	Dunkwa	22 (13.2)	145 (86.8)	167	< .001	8.6 (3.0–24.4)
	Tepa	3 (1.9)	151 (98.1)	154	0.71	1.3 (0.3–5.6)
	Allada	51 (71.8)	20 (28.2)	71	< .001	46.7 (17.5–124.8)
	Lalo	4 (30.8)	9 (69.2)	13	< .001	20.0 (5.6–71.2)
	Pobè	260 (49.2)	268 (50.8)	528	< .001	32.0 (12.1–85.0)
Age	(median (IQR))	12 (8–30)	14 (8–32)		0.39	
Sex	Male (%)	172 (31)	388 (69)	560	0.18	1.05 (0.98–1.13)
	Female(%)	170 (27)	459 (73)	629		
Ulcer						
	Yes (%)	267 (33.6)	527 (66.4)	794	< .001	1.7 (1.4–2.1)
No		74 (19.6)	303 (80.4)	377		
Functional limitation						
	Yes (%)	134 (47.5)	148 (52.5)	282	< .001	2.0 (1.7–2.4)
	No	199(23.6)	645 (76.4)	844		
Critical sites								
	Yes (%)	1 (2.7)	36 (97.3)	37	< .001	0.1 (0.0–0.6)
	No	336 (29.7)	796 (70.3)	1132		
Severe lesion**	Yes (%)	324 (40)	487 (60)	811	<0.001	1.6 (1.5–1.7)
	No (%)	18 (5)	326 (95)	344		

*Pearson chi-square, Fischer or MWU as appropriate

** WHO category II and III

In the logistic regression model, severity of the lesion was found to be a confounder, but not an effect modifier. There was no confounding by age, sex and lesion type (ulcer/preulcerative). Differences in the rate of surgical interventions remain even after adjusting for confounding by severity of the lesion and visible functional limitations at start of the treatment. Only the hospital in Tepa showed a rate of surgical interventions similar to the rate in Agogo (Table 3).

**Table 3:** Unadjusted and adjusted odds ratios for clinics.

	OR unadjusted 95% CI	OR adjusted^1^ 95% CI
Hospital		
Agogo	1	1
Dunkwa	9.7 3.3–28.7	5.6 1.8–17.4
Tepa	1.3 0.3–5.8	1.0 0.2–4.7
Allada	163.2 53.5–497.6	110.0 35.2–343.2
Lalo	28.4 6.1–132.3	33.2 6.9–159.3
Pobè	62.1 22.8–169.2	50.1 18.2–137.7

^1^ Odds ratio adjusted for severity and a visible functional limitation at start of treatment. There was no confounding by age, sex and lesion type (ulcer/preulcerative).

### Change in surgical practices over the years

**Fig 1:**
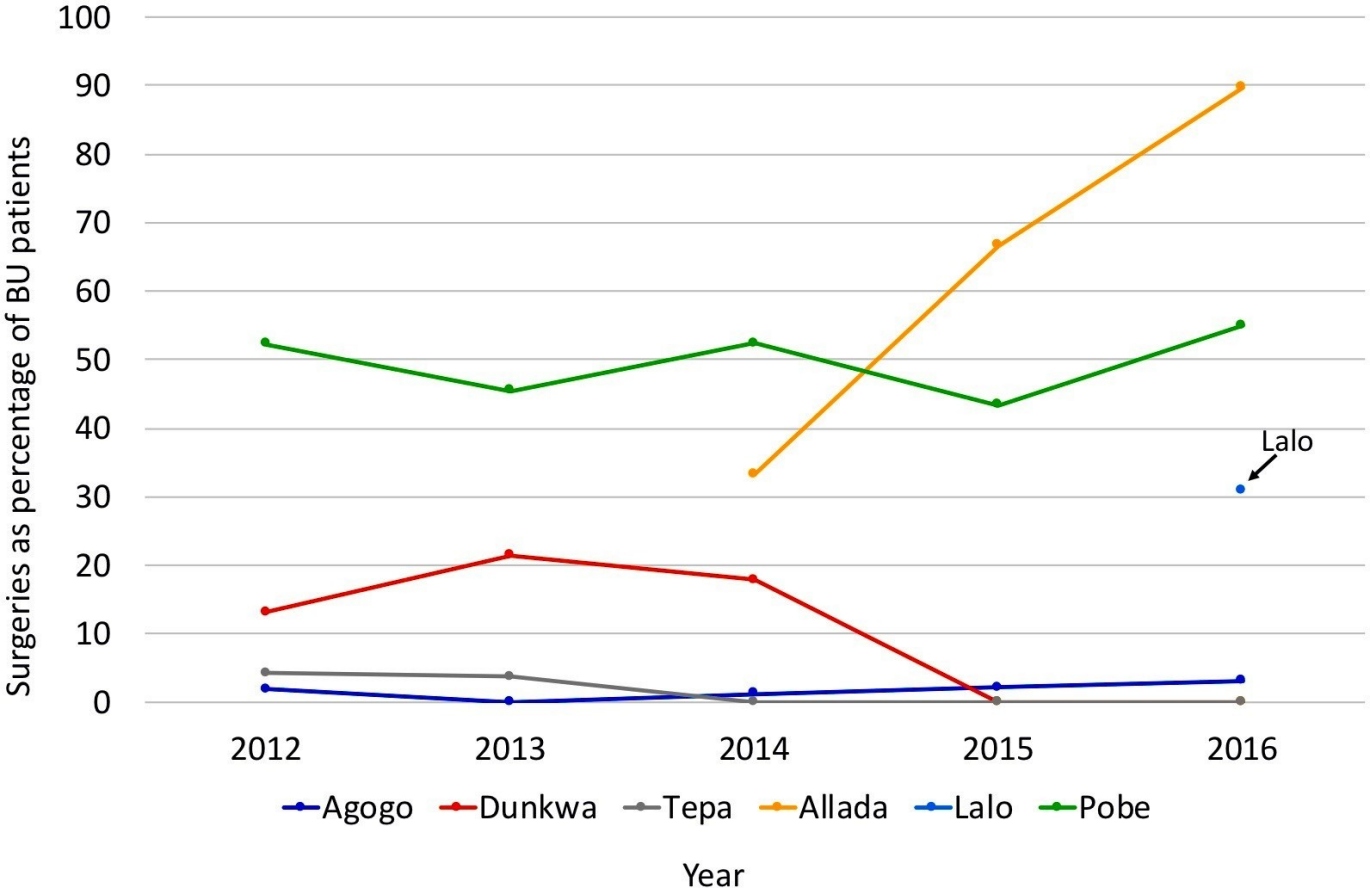
Surgical practices per center over the year. Data on surgery from Allada and Lalo not included for 2012/2013 and 2012–2015, respectively, due to clinical trial at these sites. In Allada linear by linear p< 0.001.

There was no statistically significant difference in the rate of surgical interventions over the years in the hospitals in Agogo, Tepa, Dunkwa and Pobè. In Allada the rate of surgical interventions has increased over the years (33% in 2014 to 89.7% in 2016, linear-by-linear, p< 0.001). Data on surgery from Allada and Lalo were not included for 2012/2013 and 2012–2015, respectively, due to clinical trial at these sites (Fig 1).

## Discussion

Surgery has long been the mainstay of Buruli ulcer treatment, and even with the introduction of antibiotic therapy, surgery has remained a part of Buruli ulcer treatment [3].

Despite the delay in health care seeking behavior due to fear of surgery, the costs and potential complications related to surgical intervention, there is no clear protocol in African settings for guiding the decision on the surgical procedures. Only one clinical trial has recently shown that delaying the decision to perform surgery allowed even large ulcers to heal solely with antibiotics [8]. The current study reports on the differences in decision making to perform surgery during the treatment of Buruli ulcer. Our results show that surgical practices in Buruli ulcer treatment strongly depend on the treatment center the patients present to, suggesting that it is based on divergent expert opinions.

Several studies have highlighted the relationship between severity of BU and surgical treatment [2,12,13]. However, even after controlling for the severity of the lesions, the chance of undergoing surgical intervention was still higher in the clinics of Benin. Probably, the therapeutic choices of the doctors, in these clinics, were influenced by the high number of severe cases (WHO cat II and III) received. Surgeons' opinions or enthusiasm has already been mentioned in literature as a dominant determinant of variation between areas [14]. In Ghana, on the other hand, the frequency of surgical procedures in BU patients was lower than in Benin. This observation may be explained by the fact that Buruli ulcer treatment centers in Ghana have often served as sites for clinical trials on antimicrobial treatment [7,15]. Moreover, the Tepa Government Hospital had no facility to perform surgery and had to refer patients to another hospital before surgical intervention, which may also have influenced the doctor’s decisions.

The clinical trials conducted in recent years in Benin to prove the effectiveness of postponing the decision to perform surgery [8], have not affected the habits of physicians currently performing surgery in Benin; in one treatment center the rate of surgical interventions even increased over time. Interestingly, the hospital with the increase in rate of surgical interventions, recruited new doctors, including surgeons with limited experience in Buruli ulcer treatment before working at the hospital.

The influence of the doctor's personal opinion on the decision to perform surgical intervention has often been described in the literature. A study conducted to examine surgeons’ views on the influential factors that encourage them to choose one equally fit surgical procedure over another has identified five groups of factors affecting surgeons’ decision-making: knowledge, medical condition, institutional, patient, and surgeon factors. In the absence of knowledge such as guidelines, decision-making rests largely on the remaining four factors with surgeon factors likely being the most powerful [16]. Another study suggests that surgical variation primarily reflects differences in physician beliefs about the indications for surgery and the extent to which patient preferences are incorporated into treatment decisions [17].

Surgery in West Africa where our study was conducted (Benin and Ghana), has several negative implications such as the fear of using the health services and the costs related to surgical intervention [11,18,19]. In Australia, with surgical services and a wide choice of specific antibiotics readily available, surgery plays a different role in the management of BU and indication for surgery are defined in guidelines [3,6,10]. Guidelines for the BU endemic countries in West Africa need to include the available evidence and combine the evidence with facilities available and patient preferences.

Our study is a retrospective study; there may be factors influencing doctors’ decision to perform surgery which are not included in the records.

To our knowledge, this retrospective observational study is the first to systematically investigate the influence of physician opinion on the surgical treatment of Buruli ulcer disease. A major strength of our study is that we included six hospitals from two countries and over a thousand patients for analysis, resulting in a dataset that is representative of the West African patient population. We were careful to exclude patients from Allada and Lalo who took part in the randomized clinical trial on surgical intervention in order to reduce bias on the decision to perform surgery. Nonetheless, we also acknowledge a few shortcomings. The dissemination of PCR for the diagnosis of BU has made a positive PCR result the standard scientific inclusion criterion in most studies. PCR tests were positive for the diagnosis of BU only in 82% of the included patients without being repeated if negative. We expect this to have a limited impact on the results; patients who over time received an alternative diagnosis based on the clinical evolution of the ulcer, are not included in this study. Furthermore, this study looks at the behavior of the treatment team and the rate of subsequent surgical interventions if the treatment team considers patients to have Buruli ulcer.

Our results are important for the future management of Buruli ulcer. Suggested strategies to reduce the variation of practices across clinics are practice guidelines and decision aids that have been proven effective in many clinical contexts [20]. Some authors have shown that explicit guidelines are effective in improving patient care and beneficial on practice if supported by rigorous evaluations [21,22,23]. The recent clinical trial on delaying the decision to perform surgery can be used as a basis for developing these clinical guidelines. The guidelines should include patient characteristics such as the WHO category of the lesion and the lesion position in the decision making [8]. A close collaboration between the treating physicians and surgeons remains crucial to ensure optimum use of the available evidence on the role of surgery in patient care and to prevent disabilities.

## Conclusion

Our study offers evidence for significant clinic-dependent variation in the application of surgical procedures as part of BU treatment between six hospitals from two BU endemic countries. These differences cannot be explained by differences in patient characteristics only. New strategies are needed to optimize decision making in the surgical management of BU.

## Supporting information

S1 Supporting Information STROBE checklist. (DOCX)

S2 Supporting Information Enrolment of patients included in the study (flowchart). (DOCX)
