# Supplementary material for: Buruli ulcer treatment: Rate of surgical intervention differs highly between treatment centers in West Africa
Source: PLoS Negl Trop Dis. 2019 Oct 28;13(10):e0007866. doi: 10.1371/journal.pntd.0007866 (PMC6855495; doi:10.1371/journal.pntd.0007866)
Supplement: S2 Supporting Information — (DOCX) [file pntd.0007866.s002.docx]

Supporting information 2.

Excluded

Benin : - 1 recurrent

- 227 surgical trial participants

Ghana : -10 not BU

-29 recurrent

- 4 unknown type of surgery

Total 1464

Dunkwa

167

Benin 612

Allada

71

Lalo

13

Pobè

528

Ghana 581

Tepa

154

Agogo

260

**Enrolment of patients included in the study**
